# Intravital imaging of muscle damage and response to therapy in a model of Pompe disease

**DOI:** 10.1002/ctm2.1561

**Published:** 2024-03-06

**Authors:** Naresh K. Meena, Yeap Ng, Davide Randazzo, Roberto Weigert, Rosa Puertollano, Nina Raben

**Affiliations:** ^1^ Cell and Developmental Biology Center National Heart, Lung, and Blood Institute, NIH Bethesda Maryland USA; ^2^ Intravital Microscopy Core Center for Cancer Research National Cancer Institute, NIH Bethesda Maryland USA; ^3^ Laboratory of Cellular and Molecular Biology Center for Cancer Research National Cancer Institute, NIH Bethesda Maryland USA; ^4^ Light Imaging Section Office of Science and Technology National Institute of Arthritis and Musculoskeletal and Skin Diseases, NIH Bethesda Maryland USA

Dear Editor,

Pompe disease (acid alpha‐glucosidase [GAA] deficiency) is a severe multisystem lysosomal glycogen storage disorder that is primarily marked by progressive deterioration of muscle tissues.^1^ The limited efficacy of enzyme replacement therapy (ERT) – the only available treatment option – spearheaded efforts to develop gene therapy approaches and new drugs with improved muscle targeting for ERT.^1^ Over the years, our studies using a mouse model (knockout [KO]) provided a large body of evidence indicating that: 1) the dysfunction of glycogen‐laden lysosomes leads to a major secondary abnormality in the diseased muscle – impaired autophagy and massive autophagic buildup; 2) the buildup resolution upon treatments occurs only when glycogen levels and lysosomal pool return to normal; and 3) the elimination of autophagic buildup is a reliable indicator of therapeutic efficacy.^2^, ^3^, ^4^

We have recently reported that a newly developed recombinant adeno‐associated virus (rAAV)‐based systemic gene therapy resulted in a fast and long‐lasting reversal of pathology in multiple tissues of KO mice, including the limb muscle (gastrocnemius).^2^ Preclinical testing of new therapies is a lengthy undertaking that requires a large number of animals and involves an array of techniques to analyze samples ex vivo. High‐resolution intravital microscopy (IVM), a powerful technique with a wide range of research applications, offers the possibility to visualize and quantify the effectiveness of new treatments in live animals within the natural tissue context.

In this study, we revisited the same gene therapy approach^2^ and applied IVM to peer inside living muscle cells in a reporter KO model expressing green fluorescent protein (GFP) fused to autophagosomal marker LC3. The IVM imaging of gastrocnemius muscle was performed seven weeks after a single intravenous administration of an AAV9 vector (2.5 × 10^13^ vg/kg) expressing human *GAA* transgene. The animals were 5–7 months of age at the start of therapy. (The mouse strain, referred to as GFP‐LC3:KO, and the vector are described in Supporting Information.) As expected, autophagic accumulation spanning along the longitudinal fibre axis can be seen in virtually all myofibers (94.6 ± 5.8%; *n* = 122 from three animals) of untreated GFP‐LC3:KO (Figure 1A–C, GFP‐LC3 middle panels). Consistent with our previous data,^2^ gene therapy reversed the pathology, as indicated by near complete elimination of autophagic buildup – 93.1 ±6.3 % fibres (*n* = 112 from four animals) were buildup‐free (Figure 1A–C, GFP‐LC3 bottom panels; Videos S1–S3). However, unlike in the previous study, the reversal can be directly observed in live GFP‐LC3:KO without laborious biochemical and molecular biology techniques and with a much‐reduced number of animals.

**FIGURE 1:**
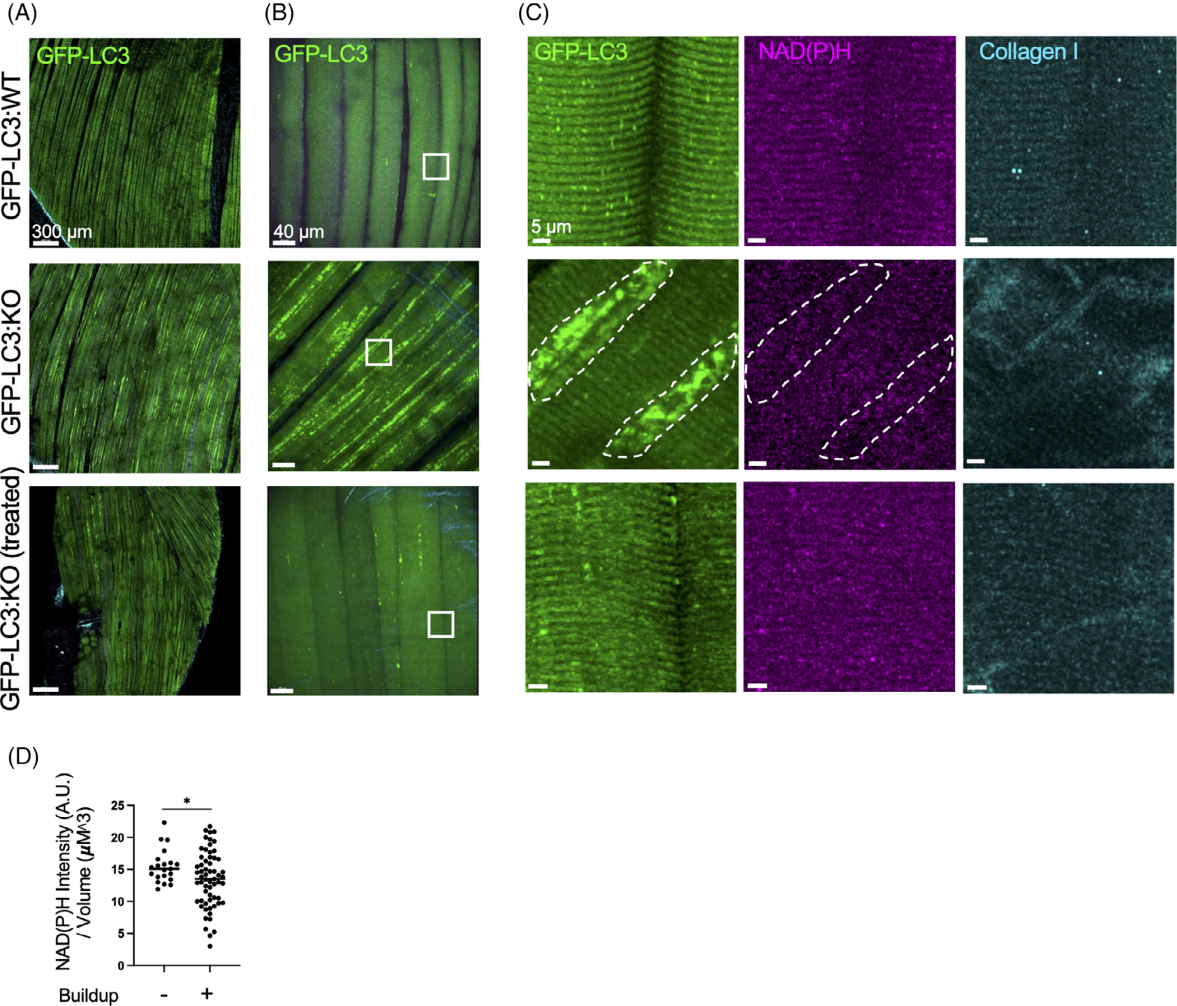
Intravital microscopy of the limb muscle of untreated and treated (gene therapy) green fluorescent protein (GFP)‐LC3:KO mice. The images show the gastrocnemius muscles of anaesthetized 5.5‐month‐old GFP‐LC3:WT (*n* = 3), 7‐month‐old untreated GFP‐LC3:KO (*n* = 3; 6–7 months of age), and GFP‐LC3:KO seven weeks after the start of gene therapy at the age of 5 months. The specimens were excited at 910 nm to visualize LC3‐GFP and collagen I (SHG), and at 740 nm to visualize NAD(P)H. (A) Large field of view; muscles were imaged in tiling mode (250 tiles, size 3743 × 5106 μm); GFP‐LC3 levels are increased only in the muscle of the untreated animal (middle panel). (B, C) Maximal projection of 42–60 μm Z stacks within the muscle layer were acquired; boxes in B mark the areas shown in C; GFP‐LC3 (green), NAD(P)H (magenta), and collagen I (SHG, cyan). The buildup areas (B) and dashed contours in (C) are observed only in the muscle of untreated mice; autophagic buildup is not seen in the muscle of treated GFP‐LC3:KO (lower panels). (D) NAD(P)H levels were quantified as described in Supporting Information and reported as AU per volume area (μm^3^, 10 field of view in three animals). Areas of autophagic buildup in the gastrocnemius muscle fibres of untreated GFP‐LC3:KO animals show reduced levels of NAD(P)H. Data presented as mean ± SD; Student's test; *p < .05.

Furthermore, we took advantage of the fact that the GFP signal can be collected together with the NAD(P)H fluorescence signal; endogenous NAD(P)H can be excited by two‐photon (2P) microscopy and used to measure mitochondrial function and metabolic activity in live animals at subcellular resolution.^5^, ^6^, ^7^

The observed NAD(P)H fluorescence intensity in the buildup areas in the muscle of GFP‐LC3:KO appeared weaker than in the neighbouring buildup‐free regions (Figure 1C). Quantitative measurements (Supporting Information) confirmed visual observations – NAD(P)H levels were significantly reduced in the buildup areas (Figure 1D), in agreement with the disruption of mitochondrial morphology/function previously reported by us and others.^8^, ^9^

Next, we explored the possibility of evaluating therapeutic efficacy by using non‐invasive imaging of the tongue muscle as a substitute for skeletal muscle (tongue involvement in Pompe patients is described in Supporting Information). This would allow for extended follow‐up to monitor the disease progression and the effect of therapies.

In preliminary experiments, we used a conventional ex vivo method – confocal microscopy of fixed single fibres isolated from the tongue of wild‐type (WT) and KO mice. Immunostaining with LAMP1 (lysosomal marker) and LC3 showed small dot‐like structures in WT fibres (Figure 2A). In contrast, LAMP1‐positive enlarged lysosomes, and blurry areas (arrows) corresponding to the buildup, were detected in the tongue muscle of KO mice (Figure 2B). LAMP1/LC3 immunostaining revealed enlarged lysosomes and lengthy areas of autophagic buildup of different shapes in KO (Figure 2C). Thus, the pathogenic mechanism in the diseased tongue muscle, much like in skeletal muscle, involves both lysosomal and autophagic pathologies.

**FIGURE 2:**
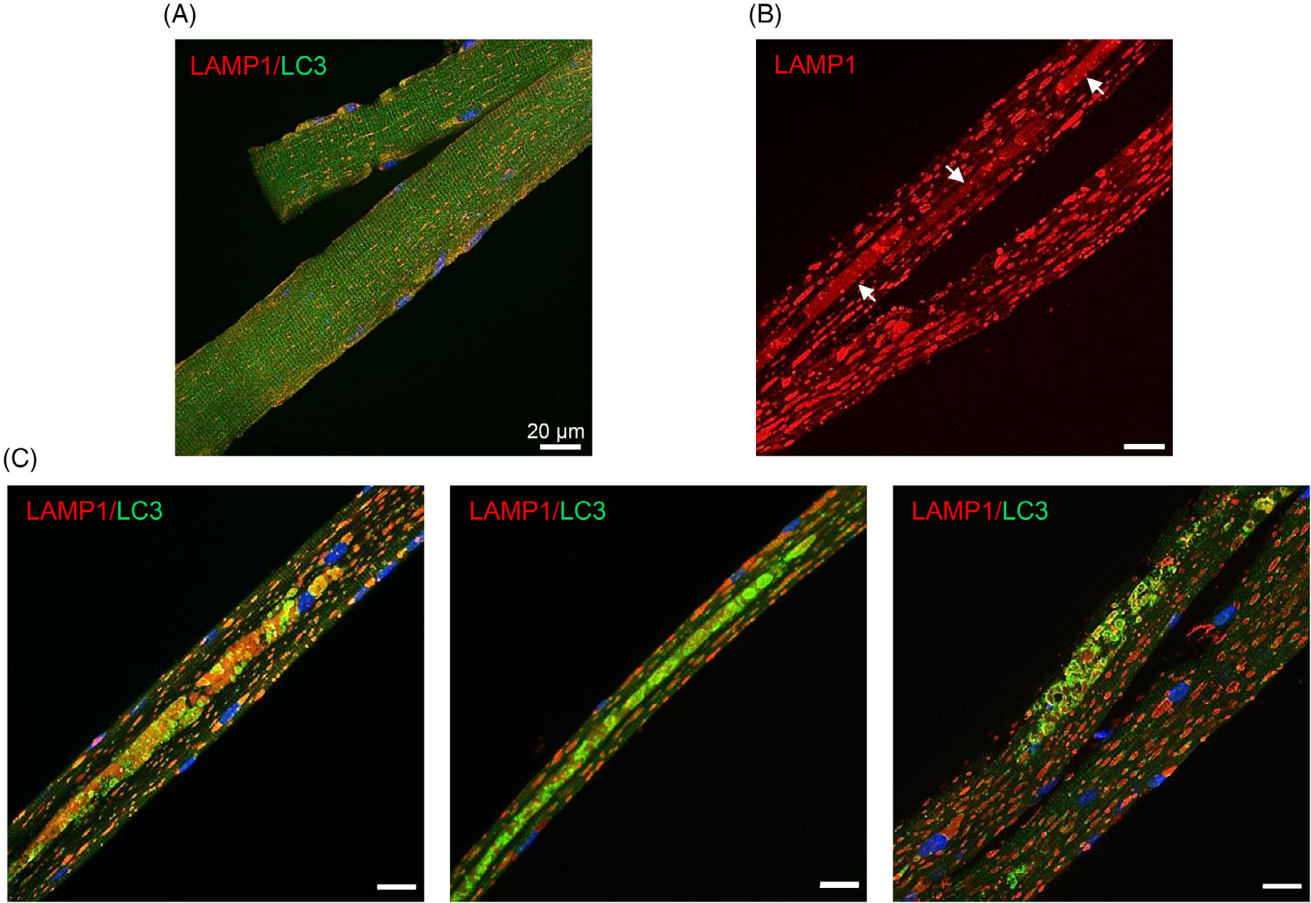
Immunostaining of single fibres from the tongue muscle of wild‐type (WT) and knockout (KO) mice. (A) Myofibers derived from WT mice (*n* = 102 fibres from two 6‐month‐old animals) were immunostained with lysosomal marker LAMP1 (Lysosome‐associated membrane protein 1) and autophagosomal marker LC3 (Microtubule‐associated protein 1 light chain 3); the image shows multiple LAMP1‐positive and LC3‐positive puncta scattered throughout the fibre; autophagic accumulation (buildup) is never seen in WT muscle. (B, C) Immunostaining of muscle fibres derived from KO mice. The image in (B) shows LAMP1‐positive enlarged lysosomes and diffuse staining (arrows) compatible with the areas of autophagic buildup. (C) Immunostaining with LAMP1 (red)/LC3 (green), and nuclei (Hoechst dye; blue); the images show enlarged lysosomes and autophagic buildup (multicoloured areas). The images are maximal projections reconstructed from five consecutive sections (Z‐stack) acquired every 1 μm (*n* = 45 fibres from 2 animals). Bars: 20μm.

IVM imaging of the tongue was performed as described in Supporting Information. The muscle layer, which is located ∼100 μm below the surface of the tongue beneath the epithelium and a layer of collagen I (Figure 3A), is well within the imaging range of 2P microscopy.^10^ First, we imaged the two‐thirds of the ventral side of the tongue at low magnification using the tiling mode to collect a large field of view (5145 × 7055 μm), which revealed bright GFP‐LC3 streaks throughout the tissue in GFP‐LC3:KO (Figure 3B; expanded images in Figure S1). Next, we acquired 42 μm z‐stacks below the collagen layer at higher magnification to visualize the myofibers (Figure 3C,D; Videos S4 and S5). Patches of GFP‐LC3, compatible in shape and distribution with autophagic buildup appeared throughout the fibres, but only in GFP‐LC3:KO. In control GFP‐LC3:WT mice (5.5‐month‐old), we observed scattered GFP‐LC3 puncta (Figure 3D, arrows), consistent with basal levels of autophagy. The NAD(P)H levels were significantly reduced in the buildup areas (Figure 3E). Analysis of age‐dependent changes revealed a slight increase in the number of fibres with autophagic buildup in 7‐month‐old compared to 3.5‐month‐old mice followed by a significant increase in 11‐month‐old animals, in which ∼80% of fibres were affected (Figure 3F).

**FIGURE 3:**
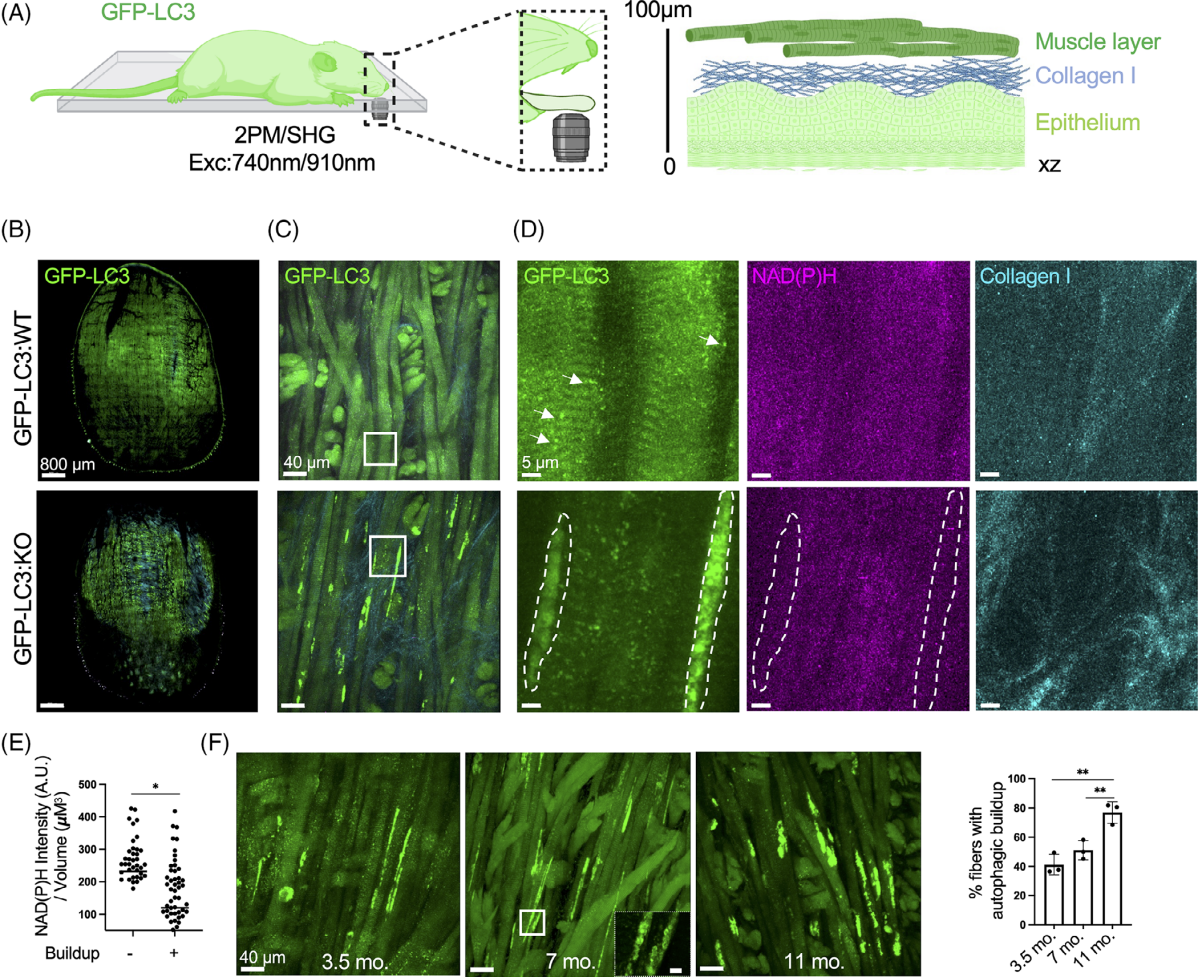
Intravital microscopy of the tongue muscle of green fluorescent protein (GFP)‐LC3:KO mice. (A) Diagram of the intravital setup for the tongue. The ventral side of the tongue of anaesthetized GFP‐LC3:WT and GFP‐LC3:KO mice was imaged as described in Figure 1. The muscle layer is located approximately 100 μm below the surface of the tongue. XZ: a cross‐sectional view of the tongue. (B–D) 7‐month‐old GFP‐LC3:KO and 5.5‐month‐old GFP‐LC3:WT were anaesthetized and imaged by two‐photon microscopy using two laser beams simultaneously (740 nm and 910 nm). (B) 2/3 of the ventral side of the tongue was imaged in tiling mode (285 tiles, size 5145 × 7055 μm, total field of view). (C, D) Maximal projection of 42 μm Z stacks was acquired within the muscle layer; boxes in (C) mark the areas shown in (D); GFP‐LC3 (green), NAD(P)H (magenta), and collagen I (SHG, cyan). (E) GFP‐LC3:KO animals were imaged as described in (C) and regions of interest (ROIs) encompassing the buildup areas (dashed contours in D) and adjacent buildup‐free areas were analyzed. NAD(P)H levels were quantified as described in Supporting Information and reported as AU per volume area (μm^3^, 10 field of view in three animals). (F) GFP‐LC3: KO animals were imaged at 3.5, 7 and 11 months of age (*n* = 3 for each group). Maximal projections of selected areas were generated, and the % of fibres exhibiting autophagic buildup was calculated (10 ROI in three animals). The inset shows ring‐shaped autophagosomes. Data in (E) and (F) presented as mean ± SD; Student's test; *p < .05; **p < .01.

As in the limb muscle, the buildup resolution was observed in the tongue muscle of GFP‐LC3:KO mice seven weeks after the start of gene therapy (Figure 4 and Figure *S2*). The vast majority of fibres (97.8 ± 1.0%; *n* = 220 from four animals) were buildup‐free. Finally, we treated 3.5‐month‐old GFP‐LC3:KO (*n* = 2) and imaged the tongue 15 weeks after dosing (Figure S2; Video 6); once again, 98.9 ± 2.2% myofibers (*n* = 81 from two animals) were buildup‐free, indicating that the therapy reversed the autophagy defect and halted its progression. Thus, the therapy was equally successful in rescuing pathology in both limb and tongue muscles.

**FIGURE 4:**
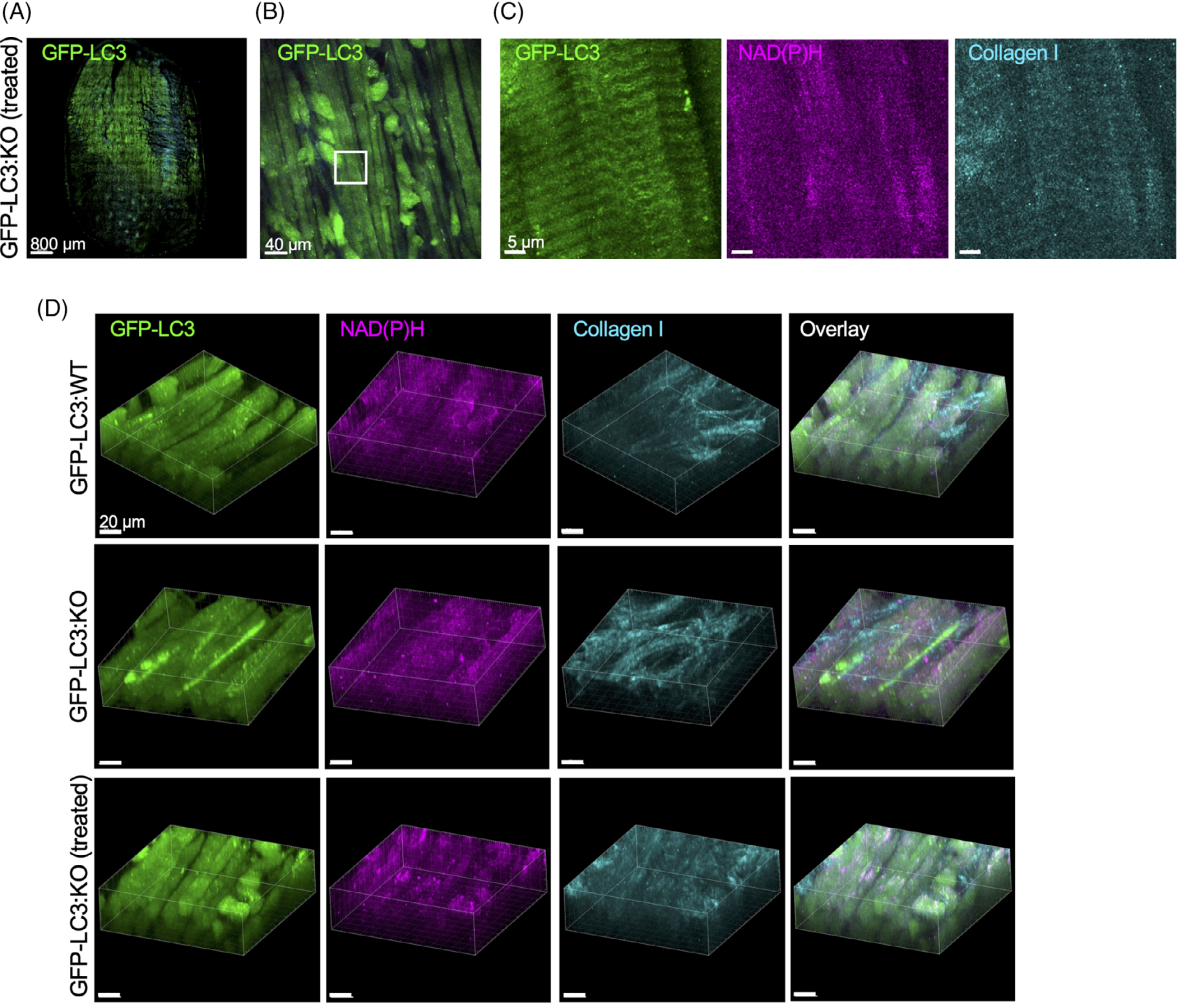
Intravital microscopy of the tongue muscle of treated (gene therapy) green fluorescent protein (GFP)‐LC3:KO mice. Top panels: The images show the tongue muscle of a GFP‐LC3:KO mouse 7 weeks after the start of gene therapy at the age of 5 months. The intravital setup and imaging were as described in Figure 3. (A) 2/3 of the ventral side of the tongue was imaged in tiling mode (285 tiles, size 5145 × 7055 μm, total field of view). (B, C) Maximal projection of 42 μm Z stacks within the muscle layer; Box in (B) marks the area shown in (C); GFP‐LC3 (green), NAD(P)H (magenta), and collagen I (SHG, cyan). No autophagic buildup was detected in >97% of fibres of treated animals. (D) Volume rendering of Z‐stacks images of GFP‐LC3:WT, untreated‐ and treated GFP‐LC3:KO mice.

In summary, this is the first report of IVM application to visualize muscle damage in Pompe disease. The reporter model enables monitoring of the disease progression and response to therapeutic interventions by non‐invasive imaging of the tongue muscle.

## AUTHOR CONTRIBUTIONS

Naresh K. Meena, Yeap Ng and Davide Randazzo performed experiments, analyzed, and interpreted the data; Roberto Weigert supervised the research and contributed to writing the manuscript; Rosa Puertollano provided funding and supervised the research; Nina Raben designed the study, performed experiments, analyzed, and interpreted the data, and wrote the paper.

## CONFLICT OF INTEREST STATEMENT

The authors declare no conflict of interest.

## ETHICS STATEMENT

All experiments were performed in accordance with the guidelines provided by the National Cancer Institute and the National Heart, Lung and Blood Institute (National Institutes of Health, Bethesda, MD, USA) Animal Care and Use Committies (ACUC) and were compliant with all relevant ethical regulations regarding animal research.

## Supporting information

Supporting Information

Supporting Information

Supporting Information

Supporting Information

Supporting Information

Supporting Information

Supporting Information

Supporting Information

Supporting Information
