# Supplementary material for: Intravital imaging of muscle damage and response to therapy in a model of Pompe disease
Source: Clin Transl Med. 2024 Mar 6;14(3):e1561. doi: 10.1002/ctm2.1561 (PMC10915738; doi:10.1002/ctm2.1561)
Supplement: Supplementary file 1 — Supporting Information [file CTM2-14-e1561-s008.docx]

**Supporting Information**

**Intravital imaging of muscle damage and response to therapy in a model of Pompe disease**

Naresh K. Meena, Yeap Ng, Davide Randazzo, Roberto Weigert, Rosa Puertollano, Nina Raben

**Materials and Methods**

*Animal Models, Treatment, and Tissue Processing*

Two animal models of Pompe disease were used: a *Gaa*^-/-^ knockout (KO) strain carrying a targeted deletion of exon 6 [1] and a reporter KO strain expressing green fluorescent protein (GFP) fused to microtubule-associated protein light chain 3 (LC3; autophagosomal marker [2]). The reporter strain (referred to as GFP-LC3:KO) was generated by crossing KO to GFP-LC3:WT mice [3] – the strain which is the most widely used to analyze autophagy in vivo. The generation of GFP-LC3:KO mice was reported previously [4]. In these animals, autophagic accumulation can be clearly seen by fluorescence microscopy as GFP-LC3-positive clusters in live myofibers isolated from different skeletal muscle groups [4]. Thus, the reporter model is an excellent tool to monitor autophagy in live Pompe mice.

In vivo efficacy of AAV9-mediated systemic gene transfer was evaluated after a single intravenous administration of AAV9.CAG.BiP.vIGF2.hGAAco.rBG.KanR vector (Amicus proprietary) into the tail vein of GFP-LC3:KO mice at a dose of 2.5x10^13^ vector genomes (vg/kg). The animals were treated for 7 and 15 weeks. Age matched untreated GFP-LC3:KO and GFP-LC3:WT were used as controls. The vector contains an engineered *hGAA* transgene that includes an insulin-like growth factor 2 (IGF2) peptide for enhanced tissue uptake and a signal peptide from BiP (the binding immunoglobulin protein) for enhanced secretion; the transgene is driven by a universal promoter (CAG; CMV early enhancer/chicken β-actin) to achieve systemic distribution among multiple tissues. In our recent publication (reference #2 in the main text), we tested three vector dosages to establish the optimal dose of 2.5x10^13^ vg/kg; the efficacy of this systemic gene transfer in skeletal muscle (as well as in the heart, diaphragm, and the CNS) was shown to result from a combination of the transgene expression in muscle, cross-correction of the surrounding non-transduced cells within the tissue, and efficient uptake of the liver-secreted IGF2-tagged hGAA circulating enzyme.

Intrinsic tongue muscle (composed of predominantly type II fibers [5]), which comprise the body of the tongue, were isolated from WT (n=2) and KO mice (n=2). Muscle fixation, isolation of single fibers, and immunostaining were performed as described [6]. Briefly, muscle strips from the resected tongue were fixed with 2% paraformaldehyde (Electron Microscopy Science)/0.1 M phosphate buffer for 30 minutes at room temperature, washed with PBS, incubated in cold methanol for 6 min at -20°C, and washed again. Single fibers were manually isolated under a dissecting microscope and stained with LAMP1 [rat anti-LAMP1 (1DB4); 1:200; BD Pharmingen, San Diego, CA) and LC3 (rabbit anti-LC3B; 1:500; Sigma–Aldrich, St. Louis, MO) antibodies using M.O.M. kit (Vector Laboratories, Burlingame, CA, USA). For each immunostaining and for confocal analysis, at least 20-25 fibers were isolated. The images were captured using a Zeiss LSM 780 confocal microscope with a pixel format of 1024 x 1024 employing an oil immersion 40X/1.4NA lens.

*Mouse preparation and experimental setup for intravital microscopy*

The noninvasive procedure for tongue imaging was performed as previously described [7]. Briefly, the mice were anesthetized by exposure to 1% isoflurane followed by an intraperitoneal injection of a mixture of ketamine (100 mg/kg) and xylazine (10 mg/kg) (Anased LA, Vet ONE) in 0.9% sterile saline solution (Quality Biological). To visualize and determine the extent of autophagic accumulation (buildup), we anesthetized the mice, gently pulled out the tongue (using blunt forceps) and stabilized it to minimize motion artifacts on the stage of a multi-photon microscope. The mouse was maintained on a heating pad throughout the procedure to maintain the body temperature at 37 °C. Throughout the procedure, the tongue was kept moist by applying carbomer 940 gel, and a gauze blanket was used to cover the mouse to maintain body temperature.

The hindlimb muscle (gastrocnemius) was surgically exposed by making a small incision of the skin and gently removing the outer connective tissue to obtain a window of ~1cm in length and ~0.5cm in width; the exposed muscle was kept moist by applying carbomer 940 gel. The mouse was then transferred to the heated stage, and a cotton stick was used to secure the leg in position.

*Image Acquisition*

The imaging was performed by using an inverted TCS SP8 Dive Spectral Microscope (Leica) equipped with Mai-Tai and Insight X3 tunable lasers (Spectral Physics), 4 spectral detectors (HyD-RLD, Leica) and a 37^o^C preheated 40X objective (NA 1.10, HC PL IRAPO, Leica). The specimens were excited at 740 nm and 910 nm simultaneously. NAD(P)H, Collagen I (Second Harmonic Generation), and GFP were detected respectively at the following emission ranges: 387-446 nm, 400-410 nm, and 504-526 nm. The tile mode of the samples was collected by bidirectional line scanning at 600 Hz (256 x 256 pixel; 12 bits per pixel) on XY and stitched using the LAS X Navigator software. The 3-D images were acquired by bidirectional line scanning with 4x line averaging at 400 Hz (512 x 512 pixel; 12 bits per pixel) on XY, and 20 to 30 Z-stacks (2 um step size) using Leica LAS X software. All the images were stored as LIF files and processed further using Imaris (Bitplane).

*Analysis of NAD(P)H signal intensity*

Mitochondrial activity in the buildup areas of muscle fibers was evaluated by means of combining signals from GFP-LC3 and the NAD(P)H endogenous fluorescence. The analysis was performed using the 3-D image rendering in Imaris. To measure the NAD(P)H signal intensity of the buildup areas, we first created IsoSurfaces based on the GFP fluorescence in the field of view using the “new surfaces” tool. The diameter threshold to mark the buildup areas was set to ≥2um. Within each sample, at least 5 IsoSurfaces were manually created in the surrounding “normal” ROIs (buildup-free regions) for comparison. The masks for these areas were transferred to the NAD(P)H channel. We then determined the NAD(P)H intensity (arbitrary unit/µm^3^) by extracting the “sum” NAD(P)H intensities of either the buildup or the buildup-free regions and dividing by the total volume (um^3^) occupied by them. A decrease in the levels of NAD(P)H suggests a reduction of metabolic activity. This could be due to i) reduction in glycolytic activity; ii) impairment in mitochondrial structure and function; or iii) both.

**Tongue involvement in Pompe disease**

Tongue abnormalities were originally attributed only to patients with the most severe infantile-onset form of Pompe disease: the consequences of glycogen accumulation in the tongue muscle –macroglossia and associated dysphagia and feeding difficulties – were documented decades ago [8] and were later shown to be nearly universal symptoms, which can manifest in infants within days after birth [9-11].

However, it is now increasingly clear that the orofacial muscles including the tongue muscle are also involved in patients with milder late-onset forms (LOPD) of the disease (reviewed in [12,13]). Clinical manifestations of the tongue muscle involvement in children and adults include macroglossia, protruding tongue, atrophy, dysphagia, and lingual weakness of different severity leading to dysarthria in some cases [14-16].The debilitating functional symptoms related to macroglossia – dysphagia, dysarthria, and sleep apnea despite the use of assisted ventilation – were recently reported in elderly patients, and some of them developed the symptoms years before the diagnosis of Pompe disease [17]. This observation is, perhaps, best illustrated by a case report of a patient who presented with progressive tongue involvement as the sole symptom; he experienced a diagnostic odyssey with multiple clinical evaluations, imaging studies, laboratory tests, and a needless treatment [18].

Different techniques have been employed to evaluate the tongue involvement in LOPD patients: manual and quantitative assessment of lingual strength [14, 16]; ultrasonography*s*howing interstitial fibrosis and fatty replacement [19]; the brain magnetic resonance imaging (MRI) showing “bright tongue sign”[20]; and whole-body MRI showing severe and consistent tongue abnormalities with intense fatty infiltration in LOPD patients [21, 22]. The tongue involvement was found to be more common in late-onset Pompe disease patients compared to those with other acquired/hereditary myopathies, suggesting that quantitative evaluation of tongue weakness may facilitate the diagnostic process [19]. Much like in humans, the tongue muscles are affected in Pompe mice [23], thus making this tissue a viable new therapeutic target. Importantly, the current enzyme replacement therapy (ERT) does not seem to alleviate the symptoms of tongue problems in Pompe disease patients [10, 11, 17].

**Statistical analysis**

Statistical significance was calculated by using GraphPad Prism software. Student's t test and one-way ANOVA were performed. Data presented as mean ± SD; p < 0.05 indicates statistical significance. The 3D images and videos were obtained using Imaris software.

**Study Approval**

All experiments using animals were performed in accordance to the guidelines provided by the National Cancer Institute (National Institutes of Health, Bethesda, MD, USA) Animal Care and Use Committee (ACUC) and were compliant with all relevant ethical regulations regarding animal research.

**References**

1. Raben N, Nagaraju K, Lee E, Kessler P, Byrne B, Lee L, et al. Targeted disruption of the acid alpha-glucosidase gene in mice causes an illness with critical features of both infantile and adult human glycogen storage disease type II. *JBC* 1998; 273(30):19086-92.

2. Kabeya Y, Mizushima N, Ueno T, Yamamoto A, Kirisako T, Noda T, et al. LC3, a mammalian homologue of yeast Apg8p, is localized in autophagosome membranes after processing. *EMBO J.* 2000;19(21):5720-8.

3. Mizushima N, Yamamoto A, Matsui M, Yoshimori T, Ohsumi Y. In vivo analysis of autophagy in response to nutrient starvation using transgenic mice expressing a fluorescent autophagosome marker. *Mol.Biol.Cell.* 2004;15(3):1101-11.

4. Spampanato C, Feeney E, Li L, Cardone M, Lim JA, Annunziata F, et al. Transcription factor EB (TFEB) is a new therapeutic target for Pompe disease. EMBO molecular medicine. 2013;5:691-706.

5. Stal P, Marklund S, Thornell LE, De Paul R, Eriksson PO. Fibre composition of human intrinsic tongue muscles. *Cells Tissues Organs*. 2003;173(3):147-61.

6. Raben N, Shea L, Hill V, Plotz P. Monitoring autophagy in lysosomal storage disorders*. Methods Enzymol.* 2009;453:417-49.

7. Amornphimoltham P, Thompson J, Melis N, Weigert R. Non-invasive intravital imaging of head and neck squamous cell carcinomas in live mice. *Methods.* 2017;128:3-11.

8. Engel AG, Gomez MR, Seybold ME, Lambert EH. The spectrum and diagnosis of acid maltase deficiency. *Neurology.* 1973;23(1):95-106.

9. Jones HN, Muller CW, Lin M, Banugaria SG, Case LE, Li JS, et al. Oropharyngeal dysphagia in infants and children with infantile Pompe disease. *Dysphagia.* 2010;25(4):277-83.

10. van Gelder CM, van Capelle CI, Ebbink BJ, Moor-van Nugteren I, van den Hout JM, et al. Facial-muscle weakness, speech disorders and dysphagia are common in patients with classic infantile Pompe disease treated with enzyme therapy. *J Inherit Metab Dis.* 2012;35(3):505-11.

11. Pena LD, Proia AD, Kishnani PS. Postmortem Findings and Clinical Correlates in Individuals with Infantile-Onset Pompe Disease. *JIMD Rep*. 2015;23:45-54.

12. Chan J, Desai AK, Kazi ZB, Corey K, Austin S, Hobson-Webb LD, et al. The emerging phenotype of late-onset Pompe disease: A systematic literature review. *Mol Genet Metab.* 2017;120(3):163-72.

13. Benz K, Hahn P, Hanisch M, Lucke K, Lucke T, Jackowski J. Systematic review of oral and craniofacial findings in patients with Fabry disease or Pompe disease. *Br J Oral Maxillofac Surg.* 2019;57(9):831-8.

14. Dubrovsky A, Corderi J, Lin M, Kishnani PS, Jones HN. Expanding the phenotype of late-onset Pompe disease: tongue weakness: a new clinical observation. *Muscle Nerve.* 2011;44(6):897-901.

15. Maggi L, Salerno F, Bragato C, Saredi S, Blasevich F, Maccagnano E, et al. Familial adult-onset Pompe disease associated with unusual clinical and histological features. *Acta Myol.* 2013;32(2):85-90.

16. Jones HN, Crisp KD, Asrani P, Sloane R, Kishnani PS. Quantitative assessment of lingual strength in late-onset Pompe disease. *Muscle Nerve.* 2015;51(5):731-5.

17. Dupe C, Lefeuvre C, Sole G, Behin A, Pottier C, Duval F, et al. Macroglossia: A potentially severe complication of late-onset Pompe disease. *Eur J Neurol*. 2022;29(7):2121-8.

18. Al-Hashel J, Ismail I. Late-Onset Pompe Disease Presenting with Isolated Tongue Involvement. *Case Rep Neurol.* 2022;14(1):98-103.

19. Jones HN, Hobson-Webb LD, Kuchibhatla M, Crisp KD, Whyte-Rayson A, Batten MT, et al. Tongue weakness and atrophy differentiates late-onset Pompe disease from other forms of acquired/hereditary myopathy. *Mol Genet Metab*. 2021;133(3):261-8.

20. Karam C, Dimitrova D, Yutan E, Chahin N. Bright tongue sign in patients with late-onset Pompe disease. *J Neurol*. 2019;266(10):2518-23.

21. Carlier RY, Laforet P, Mompoint DM, Wary C, Orlikowski D. Patterns of Muscle Involvement in Pompe Disease: A Whole-Body MRI Study. *Clin Ther.* 2010;32:S42-S3.

22. Horvath JJ, Austin SL, Case LE, Greene KB, Jones HN, Soher BJ, et al. Correlation between Quantitative Whole-Body Muscle Magnetic Resonance Imaging and Clinical Muscle Weakness in Pompe Disease. *Muscle & Nerve.* 2015;51(5):722-30.

23. Doyle BM, Turner SMF, Sunshine MD, Doerfler PA, Poirier AE, Vaught LA, et al. AAV Gene Therapy Utilizing Glycosylation-Independent Lysosomal Targeting Tagged GAA in the Hypoglossal Motor System of Pompe Mice. *Mol Ther Methods Clin Dev.* 2019;15:194-203.

**Supplemental Figures and Videos**

**Figure S1.** Expanded view of images in Figure 3B (main text). 7-month-old GFP-LC3:KO and 5.5-month-old GFP-LC3:WT were anesthetized and imaged by two-photon microscopy; 2/3 of the ventral side of the tongue were imaged in tiling mode (285 tiles, size 5145 µm x 7055 µm) to generate large field of view.

**Figure S2. Intravital microscopy of the tongue muscle of** **treated (gene therapy)** **GFP-LC3:KO mice.** Images were performed as described in Figure 3 (main text). Top panels: The images show the tongue muscle of a GFP-LC3:KO mouse 7 weeks after the start of gene therapy at the age of 7 months (n=2). Low panels show the tongue muscle of a GFP-LC3:KO mouse 15 weeks after the start of gene therapy at the age of 3.5 months (n=2). (A) 2/3 of the ventral side of the tongue were imaged in tiling mode (285 tiles, size 5145 µm x 7055 µm, total field of view). (B and C) Maximal projection of 42 µm Z stacks were acquired within the muscle layer; the boxes in B mark the areas shown in C; GFP-LC3 (green), NAD(P)H (magenta), and collagen I (SHG, cyan). No autophagic buildup was detected in >98% muscle fibers after treatment.

**Video 1**. 3D-volume representation of Z-stack images of the limb muscle (gastrocnemius) of a GFP-LC3:WT mouse. GFP-LC3 signal is seen as dot-like structures scattered throughout the fibers. The three acquired signals, GFP-LC3 (green), NAD(P)H (magenta) and Collagen I (SGH; cyan), are shown only in the first few frames.

**Video 2**. 3D-volume representation of Z-stack images of the limb muscle (gastrocnemius) of untreated GFP-LC3:KO mouse. Massive autophagic buildup is seen in virtually every fiber. The three acquired signals, GFP-LC3 (green), NAD(P)H (magenta) and Collagen I (SGH; cyan), are shown only in the first few frames.

**Video 3**. 3D-volume representation of Z-stack images of the limb muscle (gastrocnemius) of a GFP-LC3:KO mouse treated with gene therapy. No autophagic buildup is seen; the fibers look indistinguishable from those of GFP-LC3:WT mice (see Video 1).

**Video 4**. 3D-volume representation of Z-stack images of the tongue muscle of a GFP-LC3:WT mouse. GFP-LC3 signal is seen as dot-like structures scattered throughout the fibers. The three acquired signals, GFP-LC3 (green), NAD(P)H (magenta) and Collagen I (SGH; cyan), are shown only in the first few frames.

**Video 5**. 3D-volume representation of Z-stack images of the tongue muscle of a GFP-LC3:KO mouse. Autophagic buildup (GFP-LC3, green) is seen in multiple fibers. The three acquired signals, GFP-LC3 (green), NAD(P)H (magenta) and Collagen I (SGH; cyan), are shown only in the first few frames.

**Video 6.** 3D-volume representation of Z-stack images of the tongue muscle of a GFP-LC3:KO mouse treated with gene therapy. No autophagic buildup is seen; the fibers look similar to those of GFP-LC3:WT mice (see Video 4).
