# Supplementary figures and images for: Intravital imaging of muscle damage and response to therapy in a model of Pompe disease
Source: Clin Transl Med. 2024 Mar 6;14(3):e1561. doi: 10.1002/ctm2.1561 (PMC10915738; doi:10.1002/ctm2.1561)

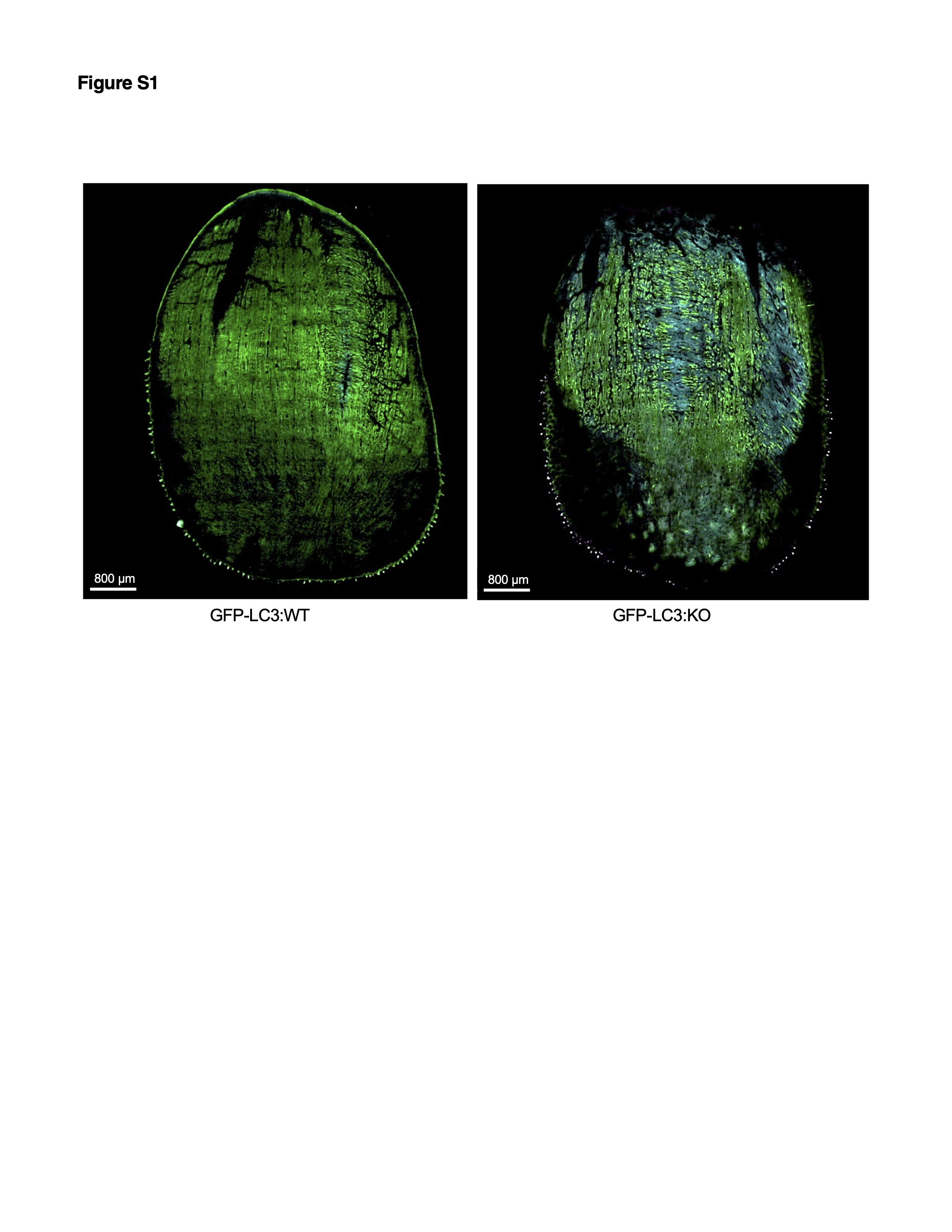

Supplement: Supplementary file 2 — Supporting Information [file CTM2-14-e1561-s006.tiff]

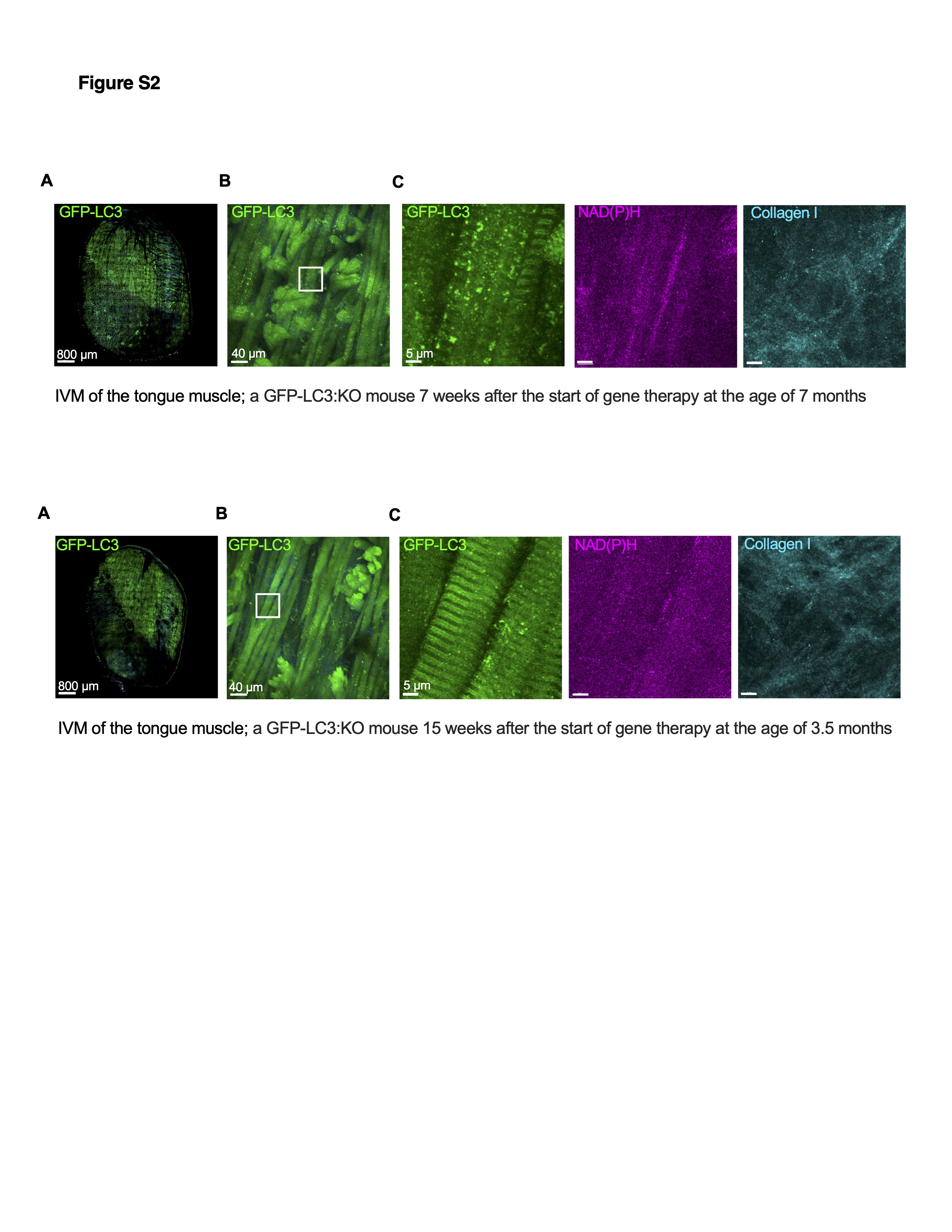

Supplement: Supplementary file 3 — Supporting Information [file CTM2-14-e1561-s003.tiff]
